# Blood urea nitrogen/creatinine ratio in heart failure: Systematic review and meta-analysis

**DOI:** 10.1371/journal.pone.0303870

**Published:** 2024-05-28

**Authors:** Yichang Zhou, Qin Zhao, Zhitong Liu, Wen Gao

**Affiliations:** 1 Cardiology Rehabilitation Department, Bayannur Hospital, Bayannur City, Inner Mongolia, China; 2 Cardiology Department, Bayannur Hospital, Bayannur City, Inner Mongolia, China; Universitas Indonesia Fakultas Kedokteran, INDONESIA

## Abstract

The meta-analysis is to evaluate the predictive value of the blood urea nitrogen / creatinine ratio (BCR) for long-term outcomes in patients with heart failure (HF). PubMed, EMBASE, the Cochrane library, and Web of Science were searched for relevant studies from inception to October 2023. STATA SE 14.0 software was used for statistical analysis. A total of 2036 reports were identified with 14 studies meeting pre-designed inclusion criteria. Three long-term outcomes were investigated. In patients with HF, the increase of BCR level indicated a greater risk of all-cause mortality (HR = 1.67, 95% CI 1.38–2.00; I2 = 90.8%, *P* = 0.000). The acute HF (AHF) subgroup demonstrated a higher risk of all-cause mortality (HR = 1.79, 95% CI 1.15–2.79; I2 = 93.9%, *P* = 0.000) as did the non-AHF subgroup (HR = 1.51, 95% CI 1.34–1.71; I2 = 37.1%, *P* = 0.122). The subgroup (≤ 70 years old) demonstrated a lower risk of all-cause mortality in patients with HF (HR = 1.62, 95% CI 1.35–1.94; I2 = 68.3%, *P* = 0.004) as did the subgroup (> 70 years old) (HR = 1.67, 95% CI 1.19–2.34; I2 = 88.3%, *P* = 0.000). In addition, this study did not support the predictive value of BCR in CVD mortality (HR = 1.48, 95% CI 0.91–2.43; I2 = 63%, *P* = 0.100) and HF hospitalization (HR = 1.28, 95% CI 0.73–2.24; I2 = 77.5%, *P* = 0.035). Sensitivity analysis showed that all the results were robust. In summary, the results showed that the blood urea nitrogen / creatinine ratio (BCR) had a significant predictive value for all-cause mortality in patients with heart failure and was a fairly promising predictor obviously. Moreover, more studies are needed to further determine the predictive value of BCR in other long-term outcomes such as CVD mortality, HF hospitalization or aborted cardiac arrest.

## Introduction

Heart failure (HF) is a clinical heterogeneous syndrome caused by ventricular filling or ejection disorder, which is manifested as fatigue, dyspnea, pulmonary edema and other symptoms [1, 2], and is often divided into acute heart failure and chronic heart failure. Epidemiological studies showed that the prevalence of HF was positively correlated with the growth of age, and the prevalence of HF in the population over 70 years old was about 10 times than that in the population under 55 years old [3]. Although the treatment of HF has made great progress in recent years, the rate of hospitalization and mortality caused by various reasons are still high in HF patients [4]. Therefore, it is necessary to spend more time looking for predictors closely related to the long-term outcomes of HF, so as to take measures to intervent as soon as possible. Whether it is acute or chronic heart failure, renal injury is one of the complications that cannot be ignored [5–7]. The decrease of cardiac contractility in patients with HF will lead to insufficient renal perfusion, and with the progression of the disease, prerenal acute kidney injury (AKI) and the irreversible loss of available nephron are predictable and inevitable [8]. These renal pathological changes indicated the obvious deterioration of HF patients and also showed a great increase in the risk of death [6]. Therefore, it is worth trying to find predictors that can predict long-term outcomes in patients with HF from clinical indicators of renal injuries.

Creatinine (Cr) and blood urea nitrogen are small molecular metabolites of nitrogen-containing substances in human body, which are representative indicators of renal function [9–11]. Clinically, the ratio of blood urea nitrogen to creatinine (BCR) is usually used to distinguish between "prerenal" renal injury caused by hypovolemia and inherent renal parenchymal diseases [12], and in patients with HF, BCR is also often used to evaluate changes of renal function caused by renal hypoperfusion due to cardiac ejection disorder [5]. Several recent studies [12, 13] believed that the increase of BCR was related to the poor prognosis of some HF phenotypes regardless of ejection fraction. It was also mentioned that this phenomenon may be related to the activation of renin-angiotensin aldosterone system (RAAS) and arginine vasopressin (AVP) system [5, 14]. In 2020, a meta-analysis [15] studied the predictive value of BCR in acute HF (AHF), but the number of included studies was limited and the meta-analysis only involved AHF patients, not all HF patients. Therefore, this study is to perform a meta-analysis to evaluate the predictive value of BCR in the long-term outcomes of patients with HF through comprehensive literature search.

## Material and methods

The present systematic review with meta-analysis was conducted according to the Preferred Reporting Items for Systematic Reviews and Meta-Analyses (PRISMA 2020) guideline [16]. This study is registered with the PROSPERO registry, number CRD42023484863.

### Search strategy

PubMed, EMBASE, the Cochrane library, and Web of Science database were comprehensively searched for relevant studies from their inception until October 2023. The study used the medical subject heading (MeSH) term of ‘plasma urea nitrogen to creatinine’ ‘BUN to Cr’ ‘Nitrogen/creatinine ratio’ ‘Cardiac Failure’ ‘heart failure’ and ‘Myocardial Failure’ as well as relevant keywords to develop the search strategy. The detailed search strategy of targeted English databases was summarized in **S1 Table in S1 File.**

### Inclusion and exclusion criteria

Inclusion criteria were as follows: 1) patients diagnosed with heart failure (HF); 2) full text written in English; 3) studies assessing the value of the blood urea nitrogen / creatinine ratio (BCR) level in predicting all-cause mortality, cardiovascular disease (CVD) mortality, HF hospitalization; 4) study design were prospective studies, retrospective studies, etc. Exclusion criteria were as follows: 1) repeated reports of the same study; 2) conference abstracts, case reports, and reviews; 3) studies with incomplete data.

### Data extraction

Eligible studies were selected by two reviewers independently, which included screening titles and abstracts and checking full texts. Disagreements between them were resolved by consulting with a third one. The following data were extracted from included studies: author’s name, publication year, country, sample size, study design, age, female%, population, BCR threshold.

### Quality assessment

The two reviewers also independently assessed the methodological strength of included studies using the Newcastle-Ottawa Scale (NOS), a procedure performed to independently assess the methodological quality of meta-analysis of observational studies [17].The assessment of NOS includes three categories of factors: (1) patient selection (three items); (2) comparability of the two study arms (two items); and (3) assessment of the outcomes (two items).Studies were awarded one point for each numbered item within the selection and exposure categories, and one or two points for comparability. Studies were graded on an ordinal scoring scale. The total points ranged from 0 to 9. Therefore, a scale of 0 to 4 points was considered poor quality, 5 to 6 points as moderate quality, and 7 to 9 points as high quality.

### Outcomes of interest

The primary focus was on long-term prognostic outcomes, encompassing all-cause mortality, cardiovascular disease (CVD) mortality, and AF hospitalization rates.

### Statistical analysis

The meta-analysis was performed using the STATA SE 14.0 software (StataCorp, College Station, Texas, USA). Hazard ratio (HR) and 95% confidence intervals (CIs) were used to assess results containing all-cause mortality, CVD mortality, HF hospitalization. Subgroup analyses were performed according to AF type, follow-up, BCR threshold, study design, or age. The study used χ2 and I-squared (I2) to evaluate the heterogeneity. The random-effect model was adopted if the p≤0.10 and I2 ≥50%, which meant existing heterogeneity among studies model. Otherwise, the fixed-effect model was applied. Publication bias was assessed using funnel plots, the Begg rank correlation [18] and egger weighted regression [19].If significant bias was present, trim-and-fill analysis was used to judge whether the publication bias had an impact on the outcomes. Subgroup analysis was used to explore possible sources of heterogeneity if necessary. Sensitivity analysis by leave-one-out method was used to test the robustness of the results. *P* < 0.05 indicated statistical significance.

## Results

### Study selection

**Fig 1:**
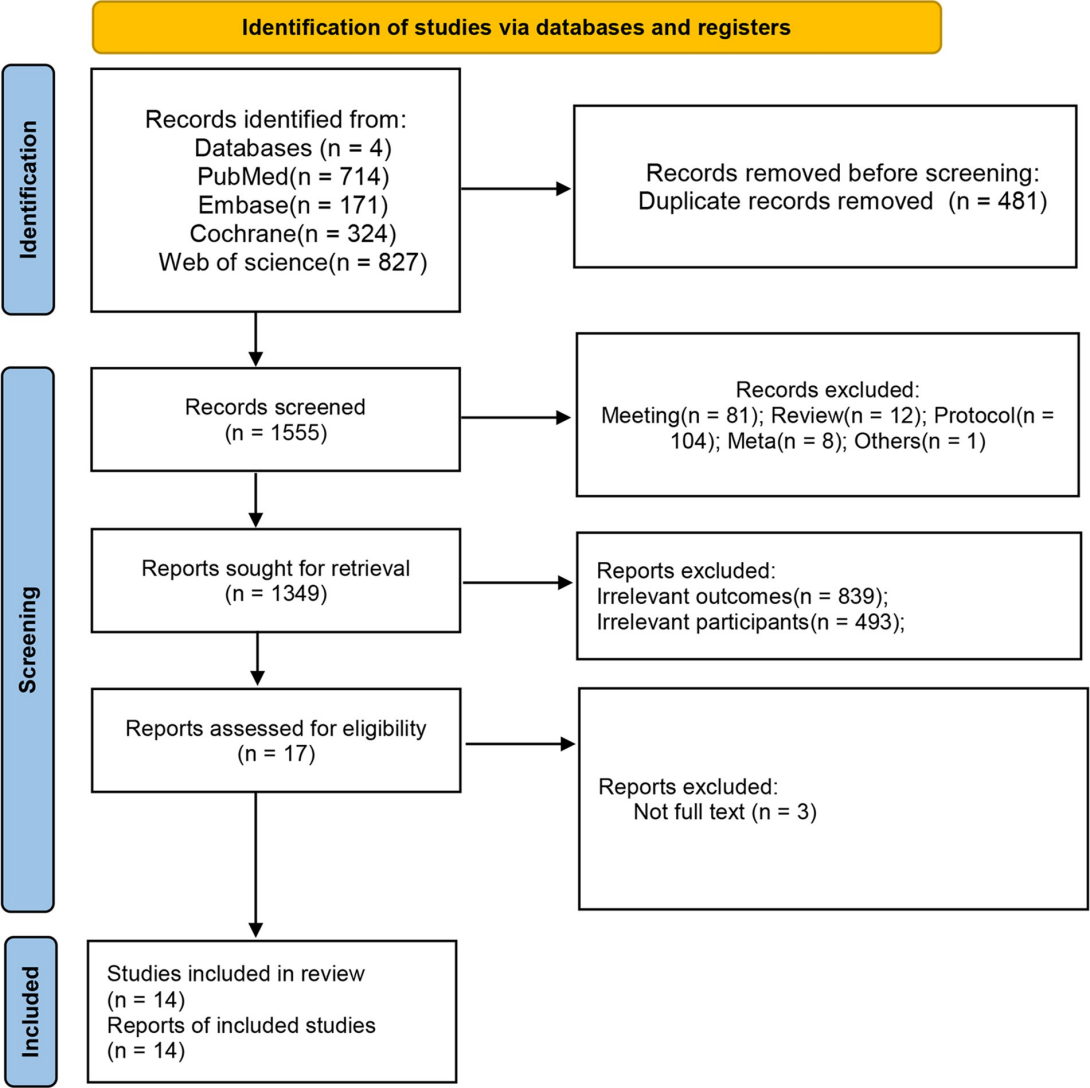
PRISMA flow chart for study screening and inclusion.

In summary, a total of 2036 studies were retrieved as potentially relevant literature reports through the initial searches in the above-mentioned databases. After the initial removal of 481 duplicate records, 1538 literatures were excluded after reviewing the title or abstract. After retrieving 17 full-length manuscripts, ultimately, 14 studies [5, 12, 14, 20–31]were eligible for data extraction and meta-analysis. The flow chart of the studies enrolled in the current study can be found in **Fig 1.**

### Study characteristics

The fourteen studies that met the inclusion criteria were published between 2004 and 2023, with sample sizes ranged from 103 to 6439. The studies were conducted in one each in the USA, China, Spain, Japan, Netherlands, and Italy. All participants were HF patients (including acute HF or chronic HF). The majority of the study population were middle or elderly age. female% ranged from 14.2 to 80.54. The BCR threshold ranged from 15.32 to 50. The participants’ demographic characteristics in the included studies can be found in **Table 1.**

**Table 1:** Baseline characteristics of 14 included studies.

Study ID	Country	Simple size	study design	Population	Age (years old)	Gender, female, n (%)	BCR threshold
Kang 2022	China	2099	retrospective study	heart failure	70	37.8	25.5
Zhen 2021	China	5121	retrospective study	heart failure	72.53	47.99	20
Otto 2017	USA	1956	retrospective study	acute heart failure	NA	NA	NA
Casado 2017 [14]	Spain	508	retrospective study	heart failure	63.19	80.54	NA
Laorden 2018	Spain	204	Prospective Study	acute heart failure	81	49.5	50
Lin 2009	China	243	Prospective Study	heart failure	63.9	35	21.1
Murata 2018	Japan	557	retrospective study	heart failure	70.15	35.19	20.4
Brisco 2017	Netherlands	6439	retrospective study	heart failure	59.4	14.2	17.3
Qian 2019	China	163	Prospective Study	acute heart failure	67.36	31.29	15.32
Wang 2023	China	504	retrospective study	heart failure	76	48.5	19.37
Aronson 2004	USA	541	retrospective study	acute heart failure	62.69	30.31	27
Brisco 2012	USA	896	retrospective study	heart failure	62.8	45.5	20
Sujino 2019	Japan	2090	Prospective Study	heart failure	76	38.1	22.1
Parrinello 2015	Italy	103	retrospective study	heart failure	74.5	56.31	25.5

**Abbreviations:** NA: not available

### Quality assessment

Newcastle-Ottawa Scales for the eligible studies were presented in **S2 Table in S1 File** and all included studies were found to exhibit an acceptable quality. Thirteen studies were evaluated as 9 points and one as seven.

### All-cause mortality

**Fig 2:**
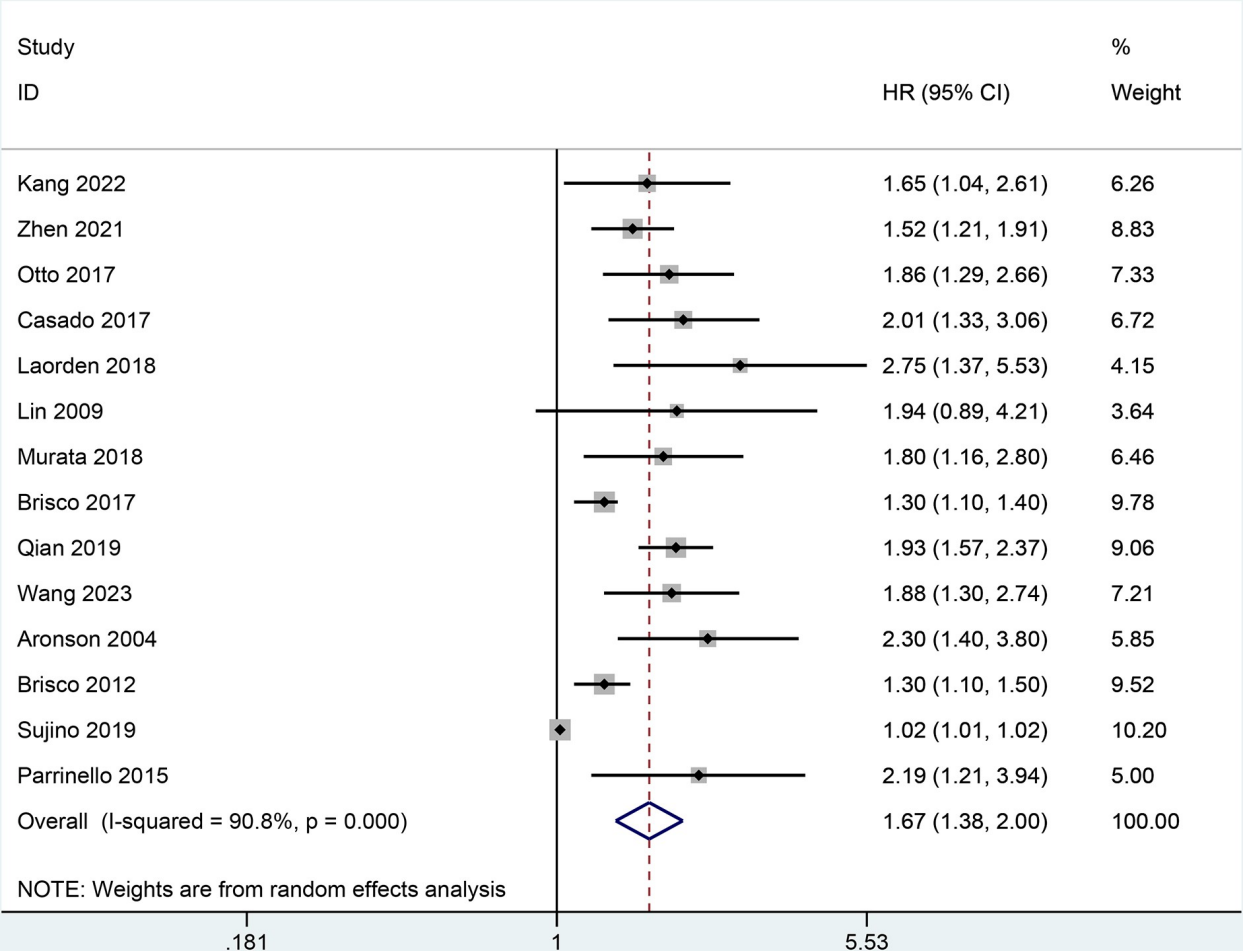
Forest plot for all-cause mortality.

Fourteen studies [5, 12, 14, 20–31]reported the value of BCR level in predicting all-cause mortality. In **Fig 2**, a random-effect model meta-analysis indicated that the increase of BCR level was potentially relevant to a higher risk of all-cause mortality in patients with HF (HR = 1.67, 95% CI 1.38–2.00; I2 = 90.8%, *P* = 0.000).

**Fig 3:**
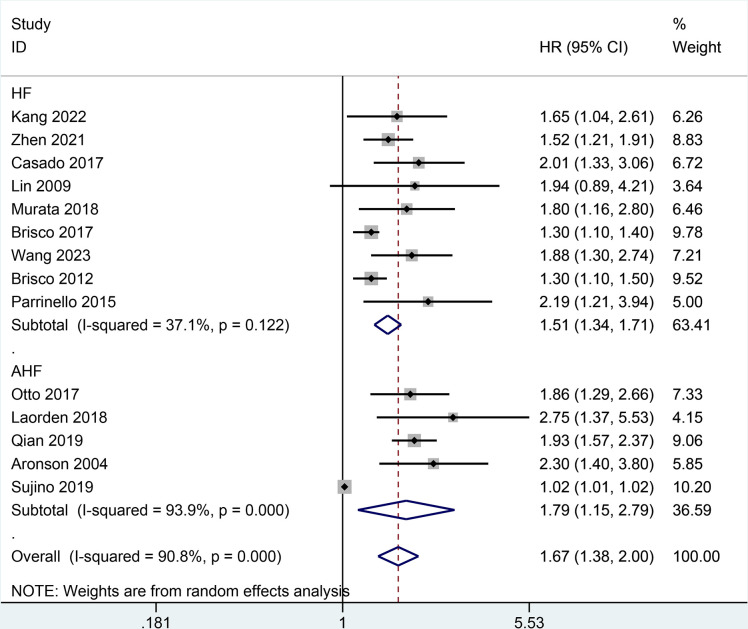
Subgroups analysis of all-cause mortality by types of HF.

**Fig 4:**
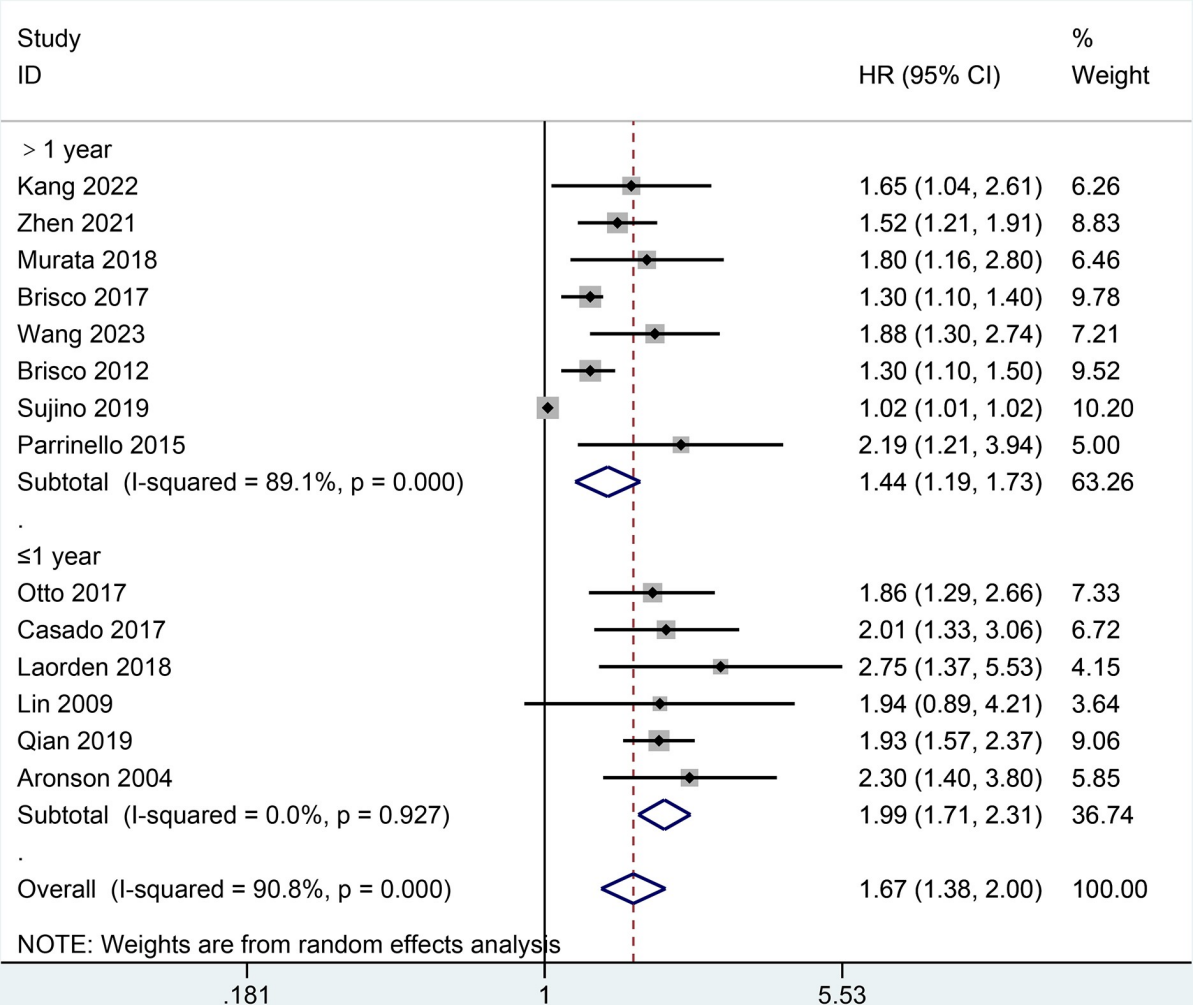
Subgroups analysis of all-cause mortality by the follow-up.

**Fig 5:**
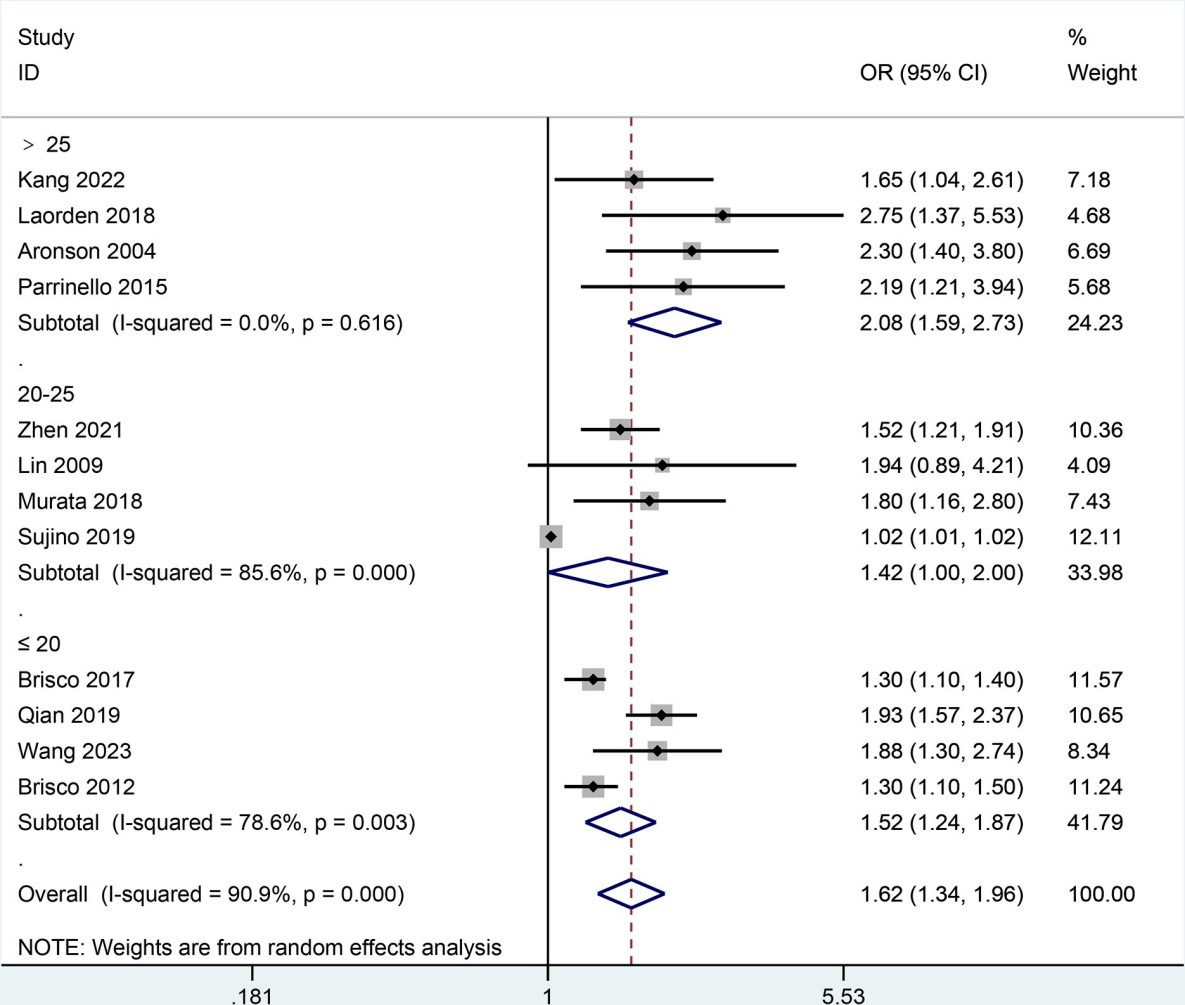
Subgroups analysis of all-cause mortality by BCR threshold.

**Fig 6:**
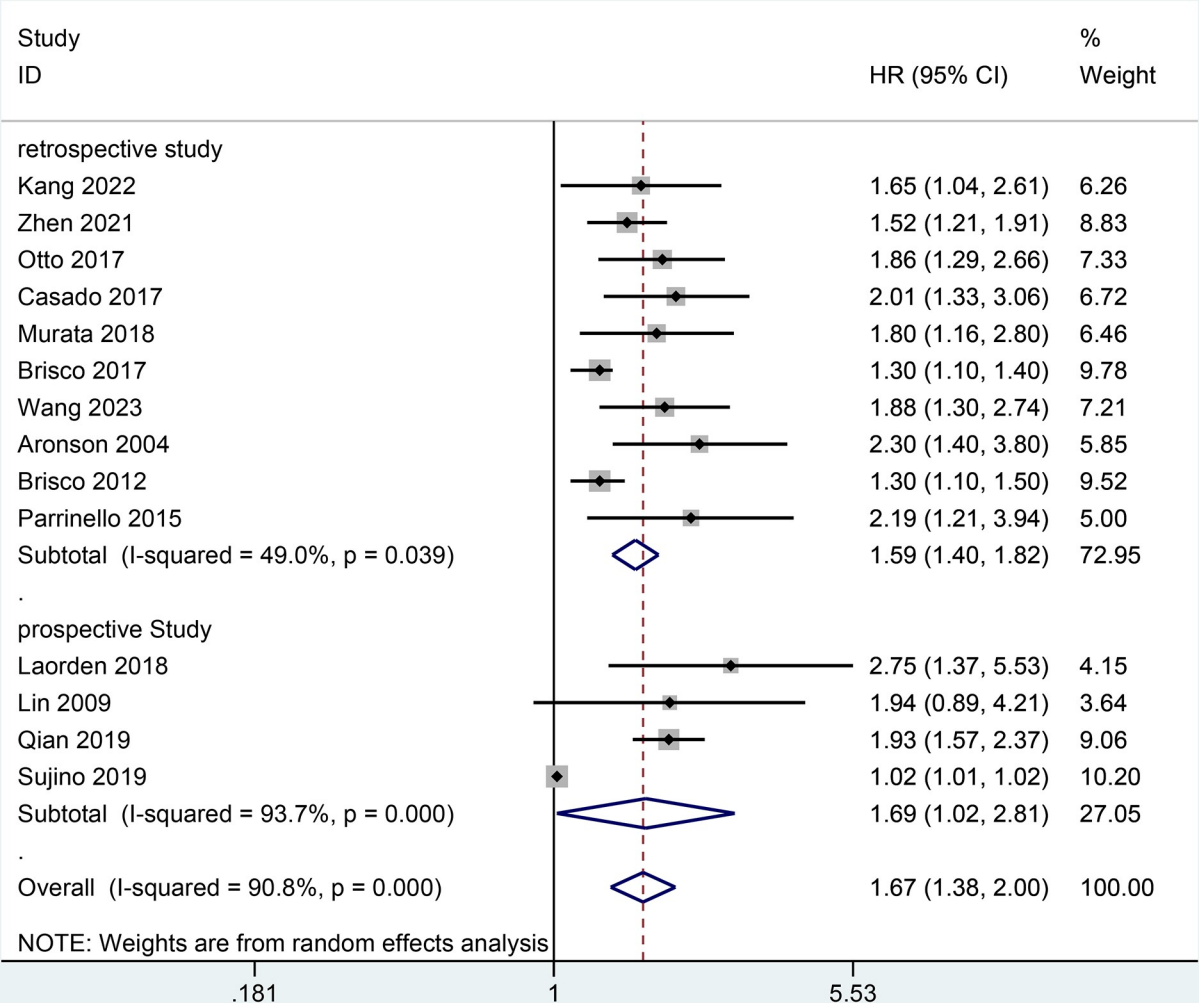
Subgroups analysis of all-cause mortality by study design.

**Fig 7:**
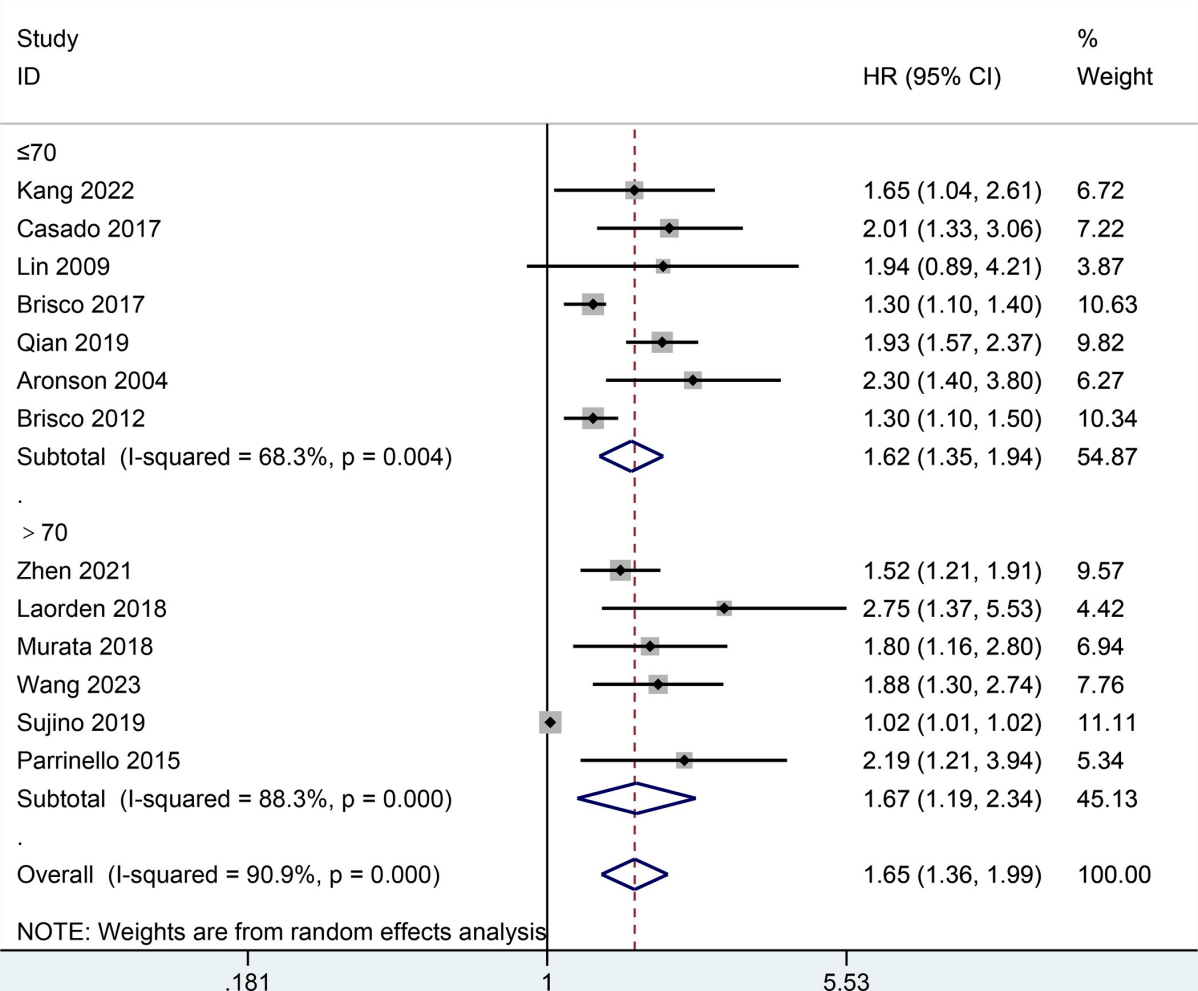
Subgroups analysis of all-cause mortality by age.

Moreover, the result of all-cause mortality was analyzed in subgroups by types of HF, follow-up, BCR threshold, study design, or age. In **Fig 3,** the AHF subgroup demonstrated that the increase of BCR level was potentially relevant to a higher risk of all-cause mortality in patients with HF (HR = 1.79, 95% CI 1.15–2.79; I2 = 93.9%, *P* = 0.000) as did the non-AHF subgroup (HR = 1.51, 95% CI 1.34–1.71; I2 = 37.1%, *P* = 0.122). In **Fig 4,** the follow-up subgroup over 1 year demonstrated that the increase of BCR level was potentially relevant to a higher risk of all-cause mortality in patients with HF (HR = 1.44, 95% CI 1.19–1.73; I2 = 89.1%, *P* = 0.000) as did the follow-up subgroup less than or equal to 1 year (HR = 1.99, 95% CI 1.71–2.31; I2 = 0.0%, *P* = 0.927). In **Fig 5,** the subgroup (the BCR threshold > 25) demonstrated that the increase of BCR level was potentially relevant to a higher risk of all-cause mortality in patients with HF (HR = 2.08, 95% CI 1.59–2.73; I2 = 0.0%, *P* = 0.616) as did the subgroup (the BCR threshold between 20 and 25) (HR = 1.42, 95% CI 1.00–2.00; I2 = 85.6%, *P* = 0.000) and the subgroup (the BCR less than or equal to 20) (HR = 1.52, 95% CI 1.24–1.87; I2 = 78.6%, *P* = 0.003). In **Fig 6,** the subgroup (the retrospective study) demonstrated that the increase of BCR level was potentially relevant to a higher risk of all-cause mortality in patients with HF (HR = 1.59, 95% CI 1.40–1.82; I2 = 49.0%, *P* = 0.039) as did the subgroup (the prospective study) (HR = 1.69, 95% CI 1.02–2.81; I2 = 93.7%, *P* = 0.000). In **Fig 7,** the subgroup (≤ 70 years old) demonstrated that the increase of BCR level was potentially relevant to a lower risk of all-cause mortality in patients with HF (HR = 1.62, 95% CI 1.35–1.94; I2 = 68.3%, *P* = 0.004) as did the subgroup (> 70 years old) (HR = 1.67, 95% CI 1.19–2.34; I2 = 88.3%, *P* = 0.000).

### Cardiovascular disease (CVD) mortality

**Fig 8:**
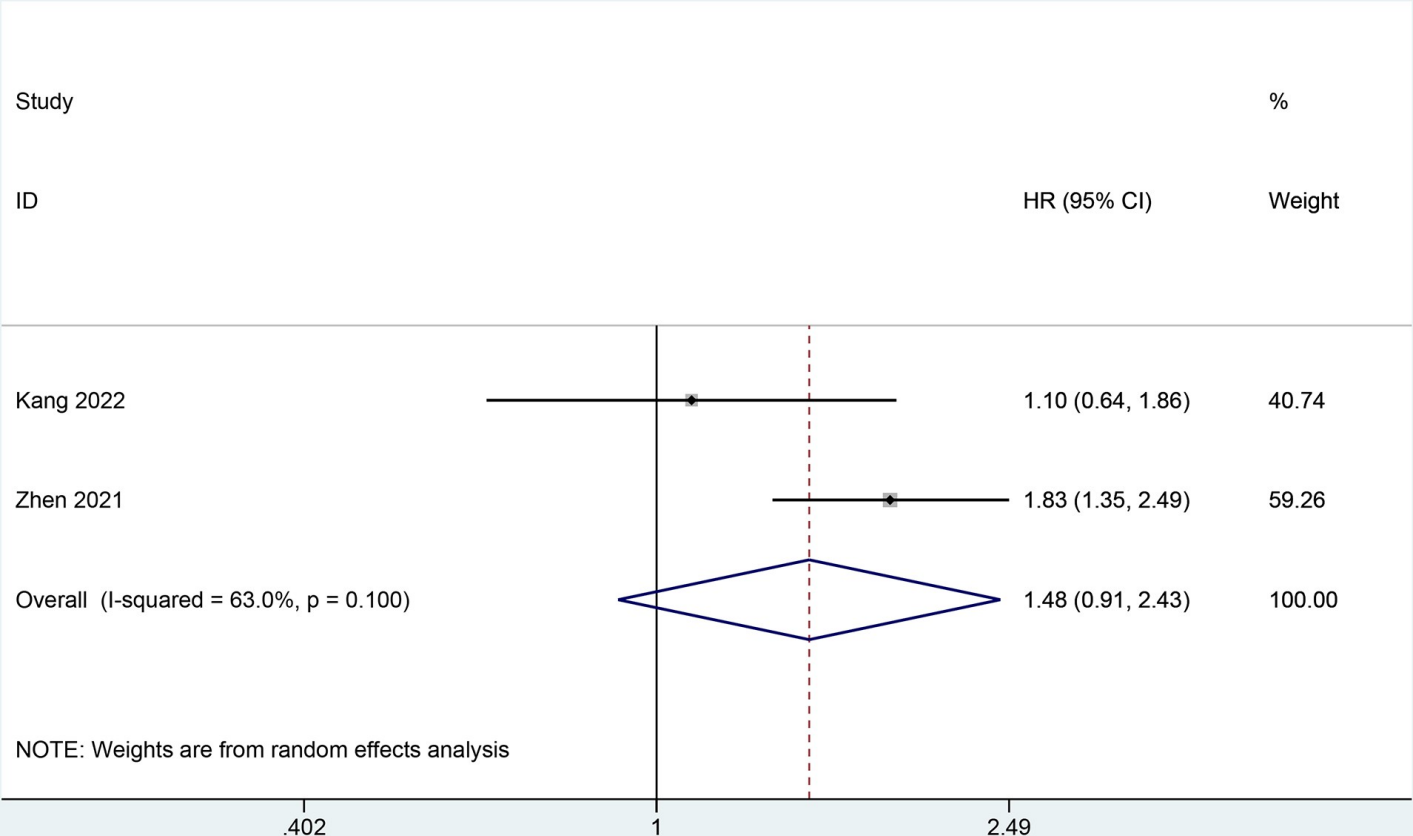
Forest plot for CVD mortality.

Two studies [5, 27]reported the value of BCR level in predicting CVD mortality. In **Fig 8**, a random-effect model meta-analysis indicated that statistically significant differences or associations was not observed (HR = 1.48, 95% CI 0.91–2.43; I2 = 63%, *P* = 0.100).

### HF hospitalization

Two studies [5, 27]reported the value of BCR level in predicting HF hospitalization. In **S1 Fig in S1 File**, statistically significant differences or associations was not observed (HR = 1.28, 95% CI 0.73–2.24; I2 = 77.5%, *P* = 0.035).

### Publication bias and sensitivity analysis

The study used the funnel plot, Begg and Egger’s test to evaluate the publication bias in this meta-analysis and no publication bias existed in all-cause mortality **(S3 Table in S1 File, S2 Fig in S1 File)**. Sensitivity analysis showed that the results of all-cause mortality were robust (**S4 Table in S1 File, S3 Fig in S1 File**). Other results were not evaluated for publication bias and sensitivity analysis because the number of included studies was limited.

## Discussion

The meta-analysis, which included a comprehensive collection of 14 studies, revealed that the BCR level had a significant predictive value for all-cause mortality in patients with HF while this study did not support the predictive value of BCR in CVD mortality and HF hospitalization. In addition, the subgroup analysis of all-cause mortality showed why the heterogeneity was so high may be relevant to the follow-up and the BCR threshold.

Clinically, the adverse clinical events that we usually discussed and related to the long-term prognosis of patients with HF included all-cause mortality, HF hospitalization, and CVD mortality [5, 12]. In recent years, it had been mentioned that BCR may provide independent and complementary prognostic information of adverse clinical outcomes, whether in acute or chronic heart failure [12, 27], and this effect would not be changed or weakened by the use of diuretics or spironolactone [5, 32, 33]. Several studies [5, 34, 35]showed that BCR and BCR variation were significantly correlated with their respective adverse clinical events after renal function stratification, and then it was concluded that BCR may not be a predictor based on renal function in patients with HF, but a neurohormonal marker for predicting adverse outcomes independent of renal function (such as RAAS, AVP). All these findings prompted us to perform this meta-analysis to explore the predictive value of BCR for the long-term outcomes of patients with HF.

All-cause mortality is the most common adverse clinical event in patients with HF [12]. In 2020, a meta-analysis [15] of prospective studies studied the predictive value of BCR for all-cause mortality in patients with AHF. Eight studies (7 from the open database and 1 from own cohort) were included in this study, and it was concluded that the highest BCR group was 77% higher than that of the lowest group in all-cause mortality. However, this study was a single-center clinical study, which did not represent all patients with HF, and the number of included studies was limited, so there was no more subgroup analysis. Therefore, our study searched the open databases and increased the number of included studies to 14. In addition, this study expanded targeted diseases to the whole HF (including AHF and chronic HF (CHF)), and made several subgroup analyses through available data. The results showed that for patients with HF, when the level of BCR increased, the risk of all-cause death increased significantly (HR = 1.67, 95% CI 1.38–2.00; I2 = 90.8%, P = 0.000), which was consistent with previous results [15]. In addition, the subgroup analysis by types of HF showed that the risk of all-cause death of AHF increased by 79% when the level of BCR increased, while that of other HF patients(non- AHF) increased by 51%, which indicated that compared with other HF patients (non- AHF), the risk of all-cause death increased more obviously in AHF patients when BCR increased. In addition, according to the subgroup analysis by follow-up, BCR threshold, study design or the age of participants, it was shown that BCR did better in predicting the risk of all-cause death in the following subgroups: follow-up (less than or equal to 1 year), BCR threshold (more than 25), study design (prospective study) and patients older than 70, that is, when BCR increased, the all-cause mortality of these groups increased by 99%, 108%, 69%, and 67% respectively. Serum creatinine and urea concentrations are influenced by multiple factors, therefore, the BCR is considered a useful parameter to reduce the aforementioned influencing factors, in evaluating the prognosis of HF patients, the BCR may be more stable and accurate than single serum creatinine and BUN in the clinical settings [36]. Our research findings may provide a favorable and relatively reliable method for guiding optimal management of HF. In addition, follow-up intervals, patients’ age should be considered in assessing the long-term outcomes for HF patients and specific measures need to be arranged based on the results from the evaluation.

In addition to all-cause mortality, HF hospitalization and CVD mortality are also the long-term outcomes that patients with HF focused on [5, 12]. This study summarized and analyzed the data of two studies, and the results showed that there was no statistically significant correlation between BCR and CVD mortality, HF hospitalization. The two results should be treated with caution because the number of included studies was limited. In addition, if enough data can be obtained in the future, the correlation between BCR and the risk of other adverse clinical events in patients with HF, such as aborted cardiac arrest, should also be analyzed.

It is necessary to consider the limitations of the present meta-analysis while interpreting the results. First, potential language bias might exist because only articles published in English were included in this literature, studies in other languages are need to address this bias. Second, the outcomes might be affected by various factors, including the follow-up, gender, age, prior comorbidities and BCR threshold, et.al. However, due to the limited sample size and available information in each study, this study failed to analyze more factors resulting in the high heterogeneity. Thirdly, the number of studies included is limited. Discussing the correlation between BCR level and CVD mortality, HF hospitalization is of great clinical significance, but the number of studies that can be included was very limited, which may be difficult to get convincing results. Fortunately, no publication bias existed in this study and sensitivity analysis showed that the pooled effect size results were robust. More well-designed studies or patient level data are warranted to comprehensively explore the potential factors that would affect the results of the pooled analyses and to verify the findings of this study in the future.

## Conclusion

The results of the study showed that the blood urea nitrogen / creatinine ratio (BCR) had a significant predictive value for all-cause mortality in patients with heart failure and was a fairly promising predictor obviously. Moreover, more studies are needed to further determine the predictive value of BCR in other long-term outcomes such as CVD mortality, HF hospitalization or aborted cardiac arrest.

## Supporting information

S1 Checklist (DOCX)

S1 File (DOCX)
