# Supplementary material for: Blood urea nitrogen/creatinine ratio in heart failure: Systematic review and meta-analysis
Source: PLoS One. 2024 May 28;19(5):e0303870. doi: 10.1371/journal.pone.0303870 (PMC11132513; doi:10.1371/journal.pone.0303870)
Supplement: S1 File — (DOCX) [file pone.0303870.s002.docx]

**Supplementary Table 1**.**Search strategy of PubMed**

| NO. | Search Details | Results |
| --- | --- | --- |
| #5 | (#1 or #2) and #3 Filter: Humans | 714 |
| #4 | (#1 or #2) and #3 | 968 |
| #3 | ((((((((plasma urea nitrogen to creatinine) OR (serum bun to creatinine)) OR (BUN to Cr)) OR (BUN to Scr)) OR (serum urea nitrogen to creatinine)) OR (Nitrogen/creatinine ratio)) OR (BUN/Cr)) OR (blood urea nitrogen to creatinine)) OR (BUN/Scr) | 17,646 |
| #2 | (((((((((((((((((((((((Cardiac Failure) OR (Heart Decompensation)) OR (Right-Sided Heart Failure)) OR (Right Sided Heart Failure)) OR (Myocardial Failure)) OR (Congestive Heart Failure)) OR (Left-Sided Heart Failure)) OR (Left Sided Heart Failure)) OR (cardiac backward failure)) OR (cardiac decompensation)) OR (cardiac incompetence)) OR (cardiac insufficiency)) OR (cardiac stand still)) OR (cardial decompensation)) OR (cardial insufficiency)) OR (chronic heart failure)) OR (chronic heart insufficiency)) OR (decompensatio cordis)) OR (heart backward failure)) OR (heart incompetence)) OR (heart insufficiency)) OR (insufficientia cardis)) OR (myocardial insufficiency)) OR (heart failure) | 404,677 |
| #1 | "Heart Failure"[Mesh] | 148,651 |

Search strategy of EMBASE

| No. | Query | Results |
| --- | --- | --- |
| #4 | (#1 OR #2) AND #3 | 171 |
| #3 | 'plasma urea nitrogen to creatinine':ti,ab,kw OR 'serum bun to creatinine':ti,ab,kw OR 'bun to cr':ti,ab,kw OR 'bun to scr':ti,ab,kw OR 'serum urea nitrogen to creatinine':ti,ab,kw OR 'nitrogen/creatinine ratio':ti,ab,kw OR 'bun/cr':ti,ab,kw OR 'blood urea nitrogen to creatinine':ti,ab,kw OR 'bun/scr':ti,ab,kw | 1474 |
| #2 | 'cardiac failure':ti,ab,kw OR 'heart decompensation':ti,ab,kw OR 'right-sided heart failure':ti,ab,kw OR 'right sided heart failure':ti,ab,kw OR 'myocardial failure':ti,ab,kw OR 'congestive heart failure':ti,ab,kw OR 'left-sided heart failure':ti,ab,kw OR 'left sided heart failure':ti,ab,kw OR 'cardiac backward failure':ti,ab,kw OR 'cardiac decompensation':ti,ab,kw OR 'cardiac incompetence':ti,ab,kw OR 'cardiac insufficiency':ti,ab,kw OR 'cardiac stand still':ti,ab,kw OR 'cardial decompensation':ti,ab,kw OR 'cardial insufficiency':ti,ab,kw OR 'chronic heart failure':ti,ab,kw OR 'chronic heart insufficiency':ti,ab,kw OR 'decompensatio cordis':ti,ab,kw OR 'heart backward failure':ti,ab,kw OR 'heart incompetence':ti,ab,kw OR 'heart insufficiency':ti,ab,kw OR 'insufficientia cardis':ti,ab,kw OR 'myocardial insufficiency':ti,ab,kw OR 'heart failure':ti,ab,kw | 402470 |
| #1 | 'heart failure'/exp | 680312 |

Search strategy of Cochrane Library

| NO. | Search deatiles | Hits |
| --- | --- | --- |
| #1 | MeSH descriptor: [Heart Failure] explode all trees | 14623 |
| #2 | (Cardiac Failure):ti,ab,kw OR (Heart Decompensation):ti,ab,kw OR (Right-Sided Heart Failure):ti,ab,kw OR (Right Sided Heart Failure):ti,ab,kw OR (Myocardial Failure):ti,ab,kw OR (Congestive Heart Failure):ti,ab,kw OR (Left-Sided Heart Failure):ti,ab,kw OR (Left Sided Heart Failure):ti,ab,kw OR (cardiac backward failure):ti,ab,kw OR (cardiac decompensation):ti,ab,kw OR (cardiac incompetence):ti,ab,kw OR (cardiac insufficiency):ti,ab,kw OR (cardiac stand still):ti,ab,kw OR (cardial decompensation):ti,ab,kw OR (cardial insufficiency):ti,ab,kw OR (chronic heart failure):ti,ab,kw OR (chronic heart insufficiency):ti,ab,kw OR (decompensatio cordis):ti,ab,kw OR (heart backward failure):ti,ab,kw OR (heart incompetence):ti,ab,kw OR (heart insufficiency):ti,ab,kw OR (insufficientia cardis):ti,ab,kw OR (myocardial insufficiency):ti,ab,kw OR (heart failure):ti,ab,kw | 49459 |
| #3 | (plasma urea nitrogen to creatinine):ti,ab,kw OR (serum bun to creatinine):ti,ab,kw OR (BUN to Cr):ti,ab,kw OR (BUN to Scr):ti,ab,kw OR (serum urea nitrogen to creatinine):ti,ab,kw OR (Nitrogen to creatinine):ti,ab,kw OR (blood urea nitrogen to creatinine):ti,ab,kw | 3092 |
| #4 | (#1 or #2) and #3 | 324 |

Search strategy of web of science

| NO. | Search deatiles | Hits |
| --- | --- | --- |
| #1 | (((((((TS=(plasma urea nitrogen to creatinine) OR TS=(serum bun to creatinine)) OR TS=(BUN to Cr)) OR TS=(BUN to Scr)) OR TS=(serum urea nitrogen to creatinine)) OR TS=(Nitrogen/creatinine ratio)) OR TS=(BUN/Cr)) OR TS=(blood urea nitrogen to creatinine)) OR TS=(BUN/Scr) | 11726 |
| #2 | ((((((((((((((((((((((TS=(Cardiac Failure) OR TS=(Heart Decompensation)) OR TS=(Right-Sided Heart Failure)) OR TS=(Right Sided Heart Failure)) OR TS=(Myocardial Failure)) OR TS=(Congestive Heart Failure)) OR TS=(Left-Sided Heart Failure)) OR TS=(Left Sided Heart Failure)) OR TS=(cardiac backward failure)) OR TS=(cardiac decompensation)) OR TS=(cardiac incompetence)) OR TS=(cardiac insufficiency)) OR TS=(cardiac stand still)) OR TS=(cardial decompensation)) OR TS=(cardial insufficiency)) OR TS=(chronic heart failure)) OR TS=(chronic heart insufficiency)) OR TS=(decompensatio cordis)) OR TS=(heart backward failure)) OR TS=(heart incompetence)) OR TS=(heart insufficiency)) OR TS=(insufficientia cardis)) OR TS=(myocardial insufficiency)) OR TS=(heart failure) | 387191 |
| #3 | #2 AND #1 | 827 |

**Supplementary Table 2 Quality assessment of included studies by NOS**

| Study | 1 | 2 | 3 | 4 | 5 | 6 | 7 | 8 | Total |
| --- | --- | --- | --- | --- | --- | --- | --- | --- | --- |
| Kang 2022 | 1 | 1 | 1 | 2 | 1 | 1 | 1 | 1 | 9 |
| Zhen 2021 | 1 | 1 | 1 | 2 | 1 | 1 | 1 | 1 | 7 |
| Otto 2017 | 1 | 1 | 1 | 1 | 1 | 1 | 0 | 1 | 9 |
| Casado 2017 | 1 | 1 | 1 | 2 | 1 | 1 | 1 | 1 | 9 |
| Laorden 2018 | 1 | 1 | 1 | 2 | 1 | 1 | 1 | 1 | 9 |
| Lin 2009 | 1 | 1 | 1 | 2 | 1 | 1 | 1 | 1 | 9 |
| Murata 2018 | 1 | 1 | 1 | 2 | 1 | 1 | 1 | 1 | 9 |
| Brisco 2017 | 1 | 1 | 1 | 2 | 1 | 1 | 1 | 1 | 9 |
| Qian 2019 | 1 | 1 | 1 | 2 | 1 | 1 | 1 | 1 | 9 |
| Wang 2023 | 1 | 1 | 1 | 2 | 1 | 1 | 1 | 1 | 9 |
| Aronson 2004 | 1 | 1 | 1 | 2 | 1 | 1 | 1 | 1 | 9 |
| Brisco 2012 | 1 | 1 | 1 | 2 | 1 | 1 | 1 | 1 | 9 |
| Sujino 2019 | 1 | 1 | 1 | 2 | 1 | 1 | 1 | 1 | 9 |
| Parrinello 2015 | 1 | 1 | 1 | 2 | 1 | 1 | 1 | 1 | 9 |

Cohort study: 1. Representativeness of the exposed cohort; 2. Selection of the non-exposed cohort; 3. Ascertainment of exposure; 4. Comparability of cohorts on the basis of the design or analysis; 5. Comparability of cohorts on the basis of the measurement; 6. Assessment of outcomes; 7. Was follow-up long enough for outcomes to occur; 8. Adequacy of follow-up of cohorts.

**Supplementary Table 3. Publication bias and heterogeneity of summarized outcomes**

| **Outcomes** | **Publication bias** | |
| --- | --- | --- |
|  | **Begg (*P* value)** | **Egger (*P* value)** |
| All-cause mortality | 0.625 | 0.736 |

Supplementary Table 4. Sensitivity analysis results for All-cause Mortality

| Study omitted | Estimate | [95% Conf. | Interval] |
| --- | --- | --- | --- |
| Kang 2022 | 1.6669519 | 1.377362 | 2.0174274 |
| Zhen 2021 | 1.6817275 | 1.384061 | 2.0434124 |
| Otto 2017 | 1.6485677 | 1.364266 | 1.9921153 |
| Casado 2017 | 1.6402973 | 1.3589406 | 1.9799066 |
| Laorden 2018 | 1.6275164 | 1.3517849 | 1.9594904 |
| Lin 2009 | 1.6558069 | 1.3721393 | 1.998118 |
| Murata 2018 | 1.6556669 | 1.3692986 | 2.001925 |
| Brisco 2017 | 1.7300094 | 1.3893974 | 2.1541231 |
| Qian 2019 | 1.6262363 | 1.3577068 | 1.9478762 |
| Wang 2023 | 1.6470745 | 1.3633043 | 1.9899111 |
| Aronson 2004 | 1.6292701 | 1.3519086 | 1.9635358 |
| Brisco 2012 | 1.7206466 | 1.3985059 | 2.1169913 |
| Sujino 2019 | 1.6890734 | 1.4797139 | 1.9280545 |
| Parrinello 2015 | 1.6400815 | 1.3596332 | 1.9783771 |

**
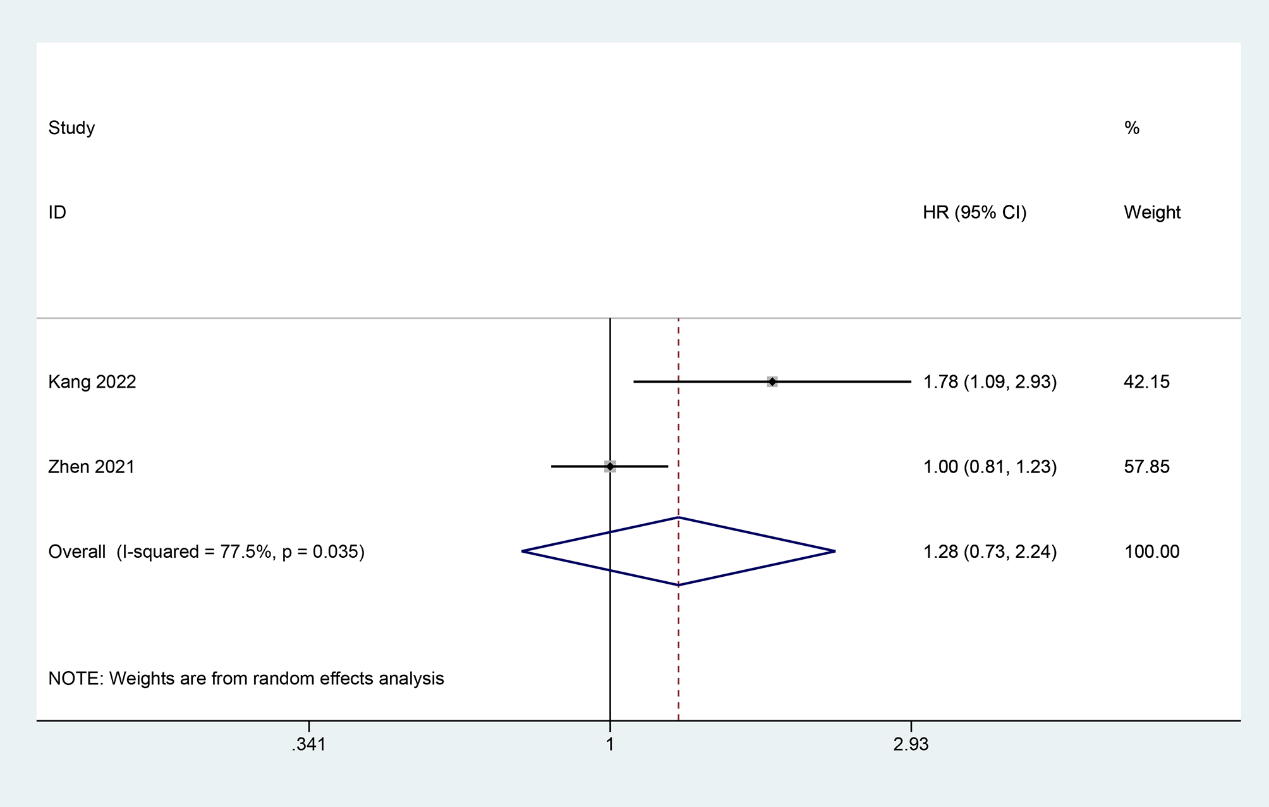
**

**Supplementary Figure 1.**Forest plot for HF hospitalization.

**
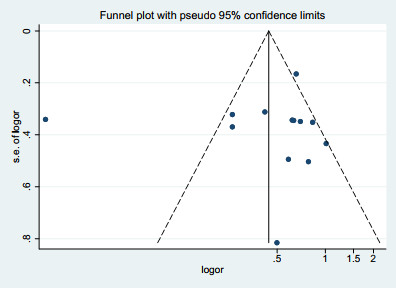
**

**Supplementary Figure 2.**The funnel plot for all-cause mortality.

**
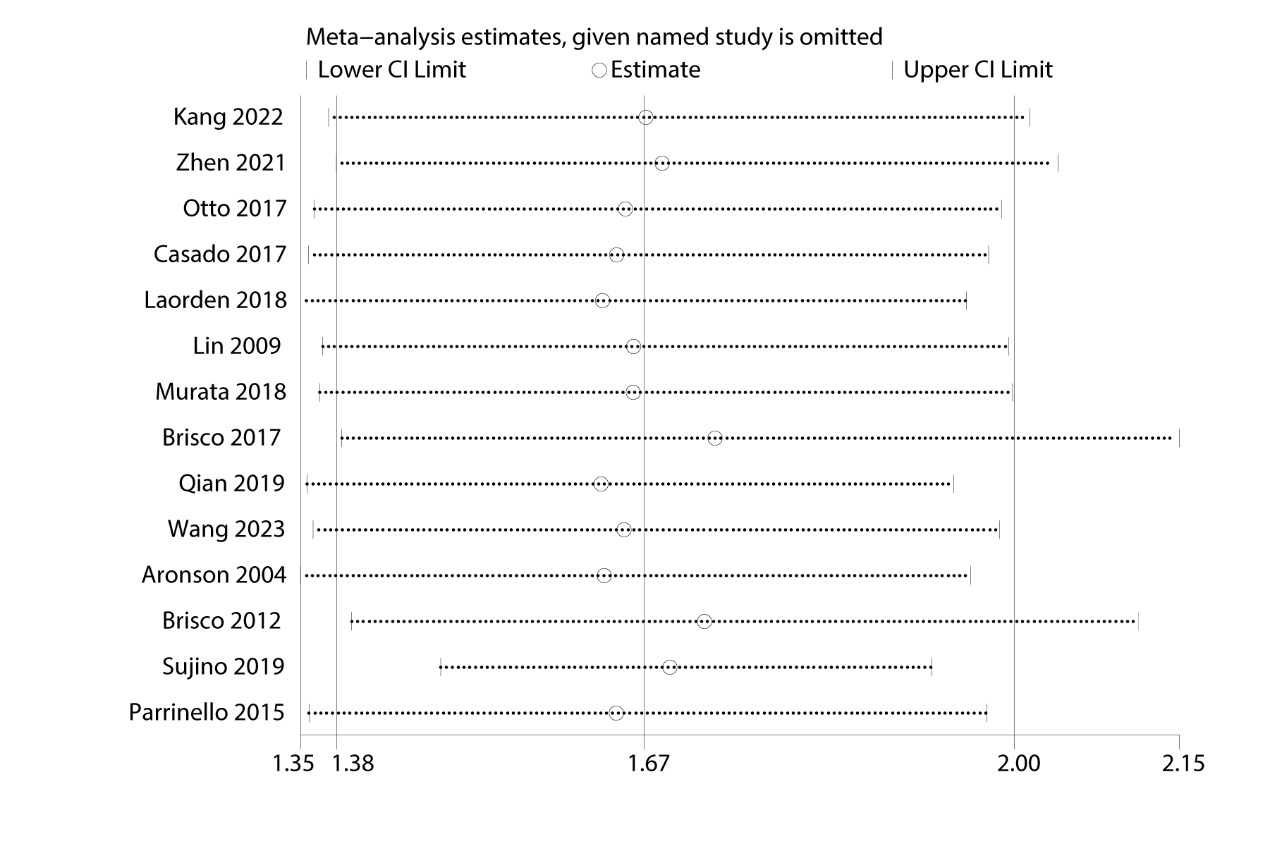
**

**Supplementary Figure 3.**Sensitivity analysis of all-cause mortality.
